# Inferring the depth of pre-instrumental earthquakes from macroseismic intensity data: a case-history from Northern Italy

**DOI:** 10.1038/s41598-019-51966-4

**Published:** 2019-10-30

**Authors:** Paola Sbarra, Pierfrancesco Burrato, Patrizia Tosi, Paola Vannoli, Valerio De Rubeis, Gianluca Valensise

**Affiliations:** Istituto Nazionale di Geofisica e Vulcanologia, Rome, Italy

**Keywords:** Natural hazards, Solid Earth sciences

## Abstract

Determining the hypocentral depth of pre-instrumental earthquakes is a long-standing geophysical issue that still awaits to be elucidated. Using very well documented recent earthquakes we found that the depth of crustal and upper-mantle events correlates well with the slope of the first 50 km of their intensity attenuation curve, regardless of their magnitude. We used this observation to build a magnitude-independent method for calculating the depth of selected historical and early-instrumental earthquakes of northern Italy based on their macroseismic intensity field. Our method relies on both standard intensity data and questionnaire-based data for 20 earthquakes, encompassing a relatively large range of magnitude (M_w_ 4.0–5.8) and depth (3.0–72.4 km), that occurred in Northern Italy between 1983 and 2019. We then used the method to estimate the depth of 20 older earthquakes that occurred in the same region between 1570 and 1972. Knowing the approximate depth of historical earthquakes is crucial for assigning them to the relevant seismogenic source, especially where seismogenic faults occur at different depths, allowing for a better characterisation of the region’s seismotectonic setting. Knowing the focal depth also allows recalculating the equivalent magnitude, which turns out to be consistently larger for deeper events, suggesting a reassessment of the local seismic hazard.

## Introduction

In this paper we discuss a new method for constraining the depth of historical earthquakes based on the attenuation of macroseismic intensity within the first 50 km of the earthquake epicentre. We show that recent, instrumentally-located earthquakes that are also well described from the macroseismic point of view can be used as a valuable key to infer the depth of past earthquakes.

**Figure 1:**
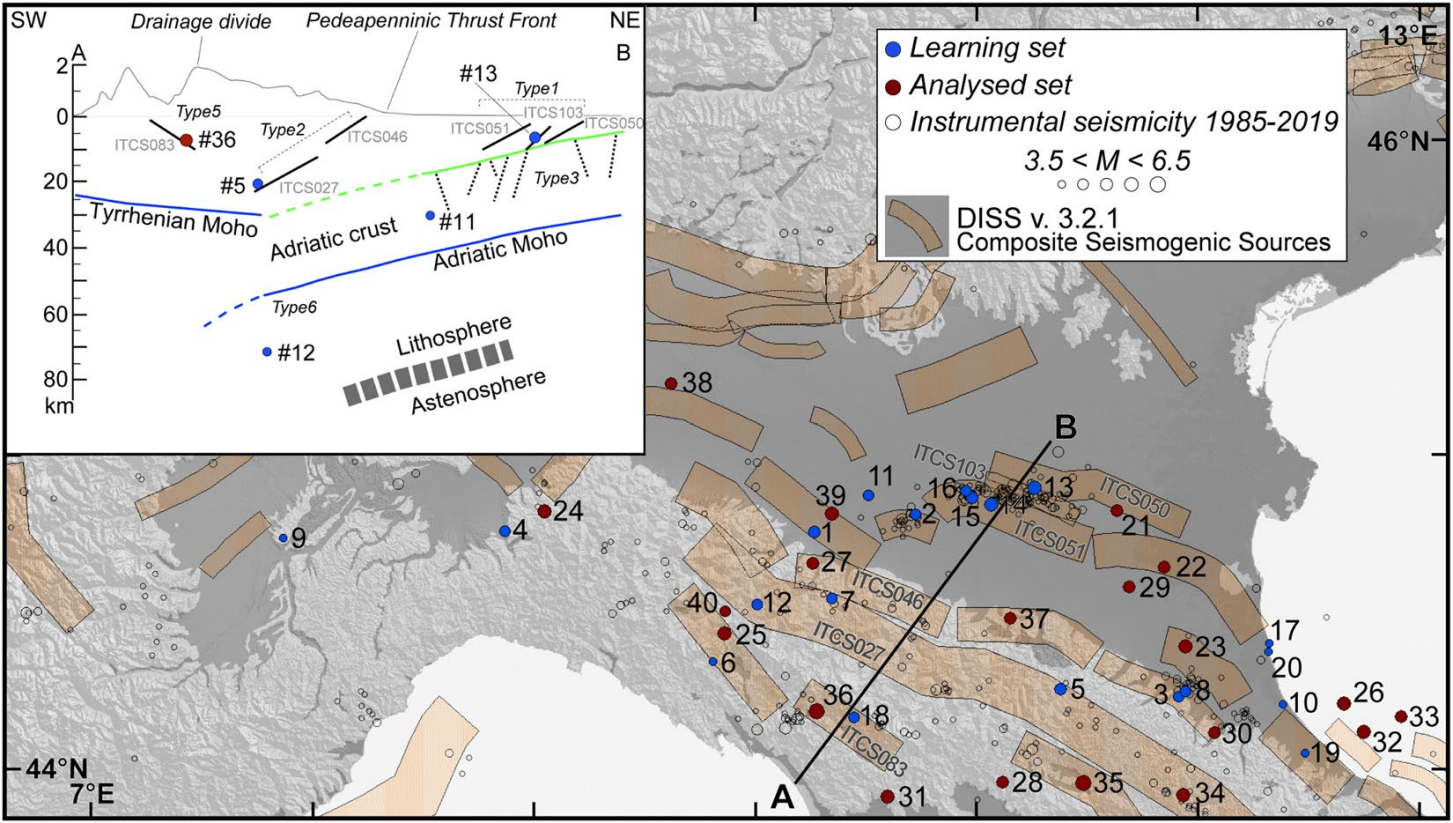
Map of the study area showing all earthquakes of M > 3.5 that occurred between 1985 and 2019 (empty circles: data from CPTI15^18^ and ISIDe^52^), all the earthquakes comprising our *learning set* (Table 2; solid blue dots), and all the analysed historical and early-instrumental earthquakes (*analysed set* in Table 3; solid red dots) for which we computed the focal depth based on the results of this work. In all cases the size of the symbols is scaled with magnitude. Orange boxes are the surface projections of Composite Seismogenic Sources from the DISS database^1^ (v. 3.2.1). The outcropping Quaternary deposits are highlighted in dark gray. (Inset) Schematic section across the Northern Apennines, showing the expected depth range and relative position of the different seismogenic source types with respect to the subduction system. The sources are plotted along with selected associated earthquakes and seismogenic sources from the DISS database. Note that the seismogenic source types 4 and 7 (Table 1) cannot be shown as they lie parallel to the strike of the section. The Moho depth is from Ziegler and Dèzes^56^; the top of the Adriatic monocline (in green) is from Livani *et al*.^13^.

Our study area includes the NE-verging Northern Apennines fold-and-thrust belt of Italy and its foredeep/foreland area in the southern Po Plain. The region is especially well-suited for our goals because local earthquakes - and hence their causative faults - exhibit largely variable kinematics, ranging from purely extensional to purely compressional, and hypocentral depth, from very shallow to deep (>100 km)^1^ (Fig. 1). It is well established that different geodynamic frameworks may be characterised by highly variable attenuation properties, ultimately resulting in rather different attenuation curves^2–7^. For example, Gasperini^2^ found that the Italian region can be subdivided into two portions - northern and southern - characterised by different attenuation properties. We maintain that the 50 km maximum epicentral distance we considered in our calculations should keep the scatter of the attenuation properties within the experimental uncertainties imposed by the nature of the observations.

The Northern Apennines thrust belt evolved within the slow Africa-Europe plate convergence; it is well described by an outer compression and inner extension pair migrating northeastward in the wake of the retreat of the subducting Adriatic slab^8,9^. The geometry of the Northern Apennines orogen is complicated by major discontinuities perpendicular to the main structural trend, interpreted as a consequence of the differential retreat of the west-dipping subduction system^10,11^.

In its turn, the Po Plain is a sedimentary basin formed by the Plio-Quaternary foredeep of the Northern Apennines^12^. Its southern portion hosts the external buried thrusts of the chain, organised in a complex system of arched fronts overlain by a variable thickness of terrigenous sediments^13^.

Besides the relatively well known, shallow active thrusts^14^ associated with the 20 and 29 May 2012, M_w_ 5.8 and 5.6, Emilia earthquakes^15,16^ and other older events, the mid- and lower-crust of the Po Plain is cut by deeper - and hence rather elusive - inherited faults; these are interpreted to have formed during the Mesozoic extensional phases preceding the inception of the Africa-Europe relative convergence^17^. There exists limited evidence for the recent activity of these faults, which may have caused some of the strongest earthquakes of the Po Plain, such as the 13 January 1909 and the 15 May 1951 events (#29 and #38 in Fig. 1). In the CPTI15 catalogue^18^ these earthquakes are currently assigned an intensity-based M_w_ 5.4 and 5.2, respectively, but their focal parameters have been recently reanalysed^1,17,19,20^.

At the rear of the compressional domain, the ongoing extension is taken up by a regional, NE-dipping, low angle normal fault system and its antithetic splays; together they form the Etrurian Fault System^1,21,22^, that is often held responsible for the relatively large 29 June 1919 and 7 September 1920 earthquakes (#35 and #36 in Fig. 1).

We assume that each seismogenic source of the study region belongs to one of seven genetically and geodynamically independent fault types, described in Table 1 and Fig. 1 and derived from a regional seismotectonic reassessment^1,20^.

**Table 1:** Seismogenic source types that exist in the study area (from Vannoli *et al*.^20^) and their estimated depth range from DISS database^1^.

Source type	Source description	Depth range (km)
1	External thrusts of the Northern Apennines	2–10
2	Internal thrusts of the Northern Apennines	10–25
3	Reactivated inherited Mesozoic faults cutting the Adriatic crust and found below the basal detachment of shallow Northern Apennines thrusts	>2
4	Lateral or relay ramps within compressional and extensional fault systems	2–30
5	Extensional faults located west of the regional drainage divide and antithetic to the subduction system and their west-dipping antithetic faults	1–10
6	Deep intraslab compressional faults	>20
7	Transverse fault systems related to differential slab retreat	>10

The depth of the source types 3, 6 and 7 is correlated to the geometry of the slab, so we provide only a minimum depth. See Fig. 1 for a conceptual sketch of their relative position.

The parameters of large earthquakes of the pre-instrumental era are a crucial piece of information for the seismotectonic characterisation of active regions and for the assessment of the associated hazard. Particularly in slow-deforming regions such as our study area, the long recurrence interval of major earthquakes makes it necessary to extend the record of ongoing tectonic activity back in time by resorting to historical information. The focal depth of such infrequent earthquakes may shed light on the thickness of the seismogenic layer and on the architecture of active faulting, thus allowing for a full 3D seismotectonic interpretation.

Unfortunately, this information is generally not supplied by current compilations^23^. In fact, the estimation of the focal depth of historical earthquakes from macroseismic data has been an unresolved issue since the early days of seismology. The problem was first tackled by Robert Mallet, an Irish engineer who is credited to have founded modern seismology. Mallet used the direction and inclination of fissures opened in the walls of buildings damaged by the 1857, M_w_ 7.1, southern Italy earthquake to infer its hypocentral depth^24^.

Later works^25,26^ modeled macroseismic data using an attenuation equation with two unknowns: the depth and the epicentral intensity. Their methodology was based on the principle that the deeper the hypocentre, the larger the area that suffers strong ground shaking. In other words, if we consider two earthquakes with the same magnitude but different hypocentral depth, the shallower event will exhibit very close isoseismal lines resembling the contour lines of steep terrain, while the contoured macroseismic field of the deeper event will resemble a gentler hilly landscape.

Several other investigators^27–32^ worked on improving the original equation; but even today, the determination of the focal depth from macroseismic data is not straightforward and requires enough macroseismic observations to allow at least three complete isoseismals to be drawn^32^. The methodology implemented by Musson in his MACDEP code^32^ consists in solving the attenuation equation for a range of values minimising the root mean square of the differences between observed and predicted intensities. Nevertheless, the depth can be obtained only when the magnitude is well-constrained by independent means, because in this approach magnitude affects the isoseismal pattern more than hypocentral depth. This methodology was applied to recent (instrumentally-recorded), low-magnitude British earthquakes illuminated by good quality macroseismic data, and the calculated depths turned out to be close to the instrumental ones^33^.

The Italian historical earthquake catalogues - CPTI15^18^ and CFTI5Med^34^ - use the Boxer code^23^ routinely for constraining the earthquake parameters. Its most recent release^35^ embeds the method proposed by Musson to determine the hypocentral depth, but relies on individual Macroseismic Data Points (MDPs) rather than on isoseismals, which makes it more reliable and less subjective. Nevertheless, a comparison between instrumental and intensity-based focal depths of Italian earthquakes shows that in most cases the two estimates are very different^35^. On these grounds the hypocentral depths were not considered reliable, and were not supplied in the latest versions of both CPTI15 and CFTI5Med.

We believe that the reason why the method developed by Musson^32^ does not seem to work on Italian earthquakes lies in the larger structural diversity of the Italian peninsula compared to Great Britain. Nevertheless, we maintain that there exists a simpler but robust macroseismic method for estimating the hypocentral depths even in extremely heterogeneous crustal regions. The method we propose rests on three fundamental pillars:

- it relies only on the slope of the attenuation curve, not on the absolute intensity levels attained by any given earthquake: as such, it is fully independent of its magnitude;
- it is based on individual MDPs, not on isoseismals: as such, it eliminates the subjectivity relating to the style of isoseismal drawing and to the associated geographic uncertainties;
- it is primarily based on data gathered through the web-based questionnaires collected in the HSIT database (Hai Sentito Il Terremoto, or *Did You Feel the Earthquake*; http://www.haisentitoilterremoto.it/): a nationwide, community-based effort for collecting information from people who felt an earthquake and for creating large and homogeneous pseudo-intensity datasets^36^. HSIT contains data relating to Italian earthquakes that have occurred since June 2007; for pre-2007 earthquakes we used data from DBMI15^37^ and from the INGV Macroseismic Bulletin^38^.

## Results

Using a set of recent, well-recorded crustal and upper-mantle earthquakes we found that their depth correlates well with the slope of the first 50 km of the curve describing the decay of macroseismic intensity with epicentral distance. The retrieved depth can then be used for constraining two equally important and totally independent aspects of seismic hazard assessment at any scale: (a) the assignment of any given earthquake to a specific seismotectonic process, i.e. to a specific source type; and (b) the re-evaluation of earthquake magnitude for all events that turn out to be deeper than “upper crustal”, assumed as the standard depth by Boxer.

The subduction system that includes the Northern Apennines fold-and-thrust belt comprises different seismogenic sources located at different depth (Table 1 and Fig. 1). Assigning each of them to the relevant seismotectonic domain is a pre-requisite of any fault-based seismic hazard assessment scheme. This step, however, requires that the focal depth be estimated with an accuracy that is smaller than the average depth difference between the different fault types coexisting in the same area. Since the earthquake attenuation is also a function of depth, the availability of this latter parameter may be the key for correctly interpreting the variability of the attenuation parameters in the different existing geodynamic settings^35^.

**Figure 2:**
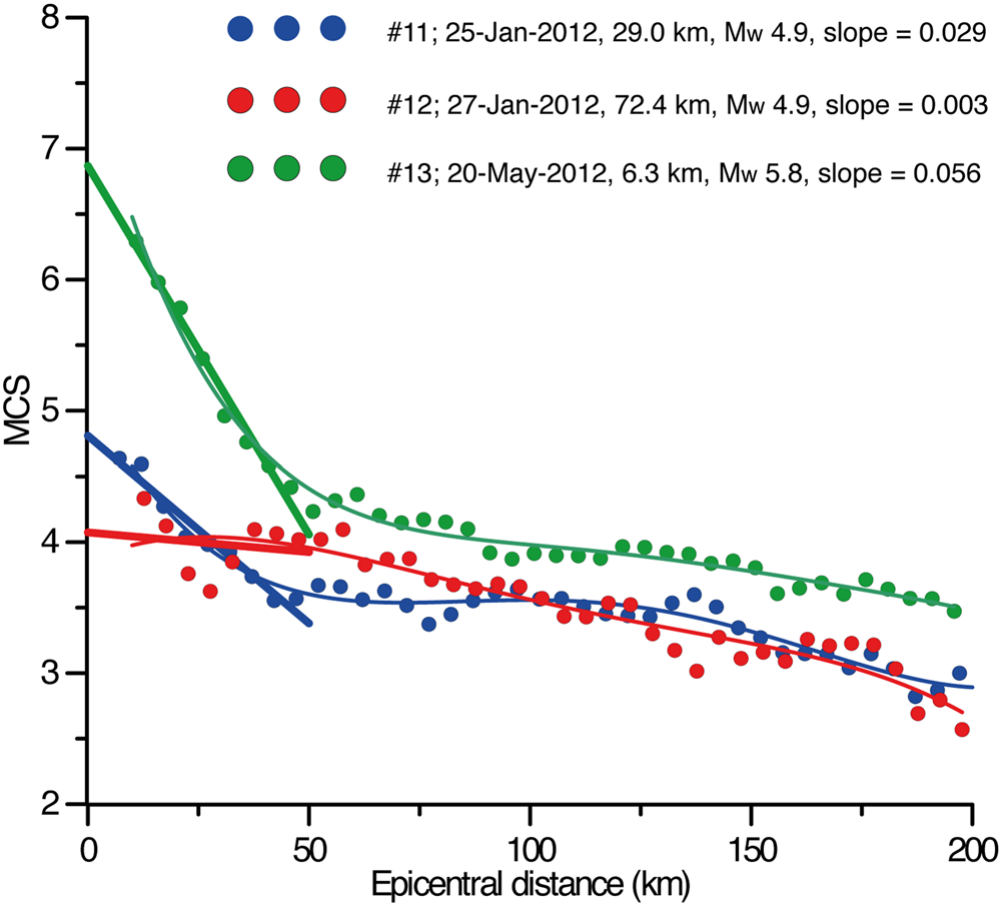
Attenuation curves obtained for three selected earthquakes (#11, #12 and #13 in Fig. 1 and Table 2). Individual intensity datapoints were obtained averaging the intensity values over a circular moving window of 10 km, overlapping the adjacent window by 5 km. We used a linear fit for the first 50 km of the curves and calculated the resulting slope.

We started off from the analysis of three instrumental earthquakes that occurred in 2012 in our study area (Fig. 2, #11, 12 and 13 in Table 2), with M_w_ ranging between 4.9 and 5.8. All three shocks were felt over a large portion of central and northern Italy, as testified by the many data reported in HSIT (an average of 9,000 observations per shock). They occurred closely spaced in time within a region where events of this size are relatively rare: yet, they exhibit a widely different focal depth, suggesting they were generated by independent tectonic processes.

**Table 2:** List of the earthquakes used as *learning set* and shown in Fig. 1.

ID	Event date	Time UTC	M	Lon	Lat	Depth (km)	Source of depth value	MDP	Total responses	Macroseismic data source	Slope	R^2^	Recalculated M_w_	Source type
1	09-Nov-1983	16:29:52	5.0	10.27	44.76	18.0	CSTI1.1	850	NA	CFTI5Med	0.039	0.97	5.3	3/7
2	02-May-1987	20:43:53	4.7	10.69	44.81	3.0	ISIDe	802	NA	DBMI15	0.065	0.97	4.5	4
3	﻿10-May-2000	16:52:11	4.8	11.93	44.24	13.1	ISIDe	151	NA	DBMI15	0.069	0.96	/	1
4	11-Apr-2003	09:26:57	4.8	8.87	44.76	8.2	ISIDe	694	NA	Gasparini *et al*. 2011	0.049	0.81	/	4
5	14-Sep-2003	21:42:53	5.2	11.38	44.26	20.1	Piccinini *et al*.^57^	133	NA	DBMI15	0.037	0.98	/	2
6	26-Mar-2008	09:19:30	4.0	9.81	44.34	72.2	ISIDe	39	85	HSIT	0.017	0.71	/	6/7
7	23-Dec-2008	15:24:22	4.9	10.35	44.54	22.9	ISIDe	670	1,712	HSIT	0.038	0.90	5.0	2
8	05-Apr-2009	20:20:53	4.5	11.91	44.23	24.5	ISIDe	368	1,389	HSIT	0.020	0.66	3.8	2/3
9	19-Apr-2009	12:39:50	4.1*	7.87	44.74	45.3	ISIDe	384	1,650	HSIT	0.013	0.97	4.2	7
10	13-Oct-2010	22:43:14	4.0	12.38	44.21	26.5	ISIDe	175	1,304	HSIT	0.024	0.80	3.7	7
11	25-Jan-2012	08:06:37	4.9	10.51	44.87	29.0	ISIDe	1,354	5,851	HSIT	0.029	0.96	4.9	3
12	27-Jan-2012	14:53:13	4.9	10.01	44.52	72.4	ISIDe	1,547	7,798	HSIT	0.003	0.03	5.2	6
13	20-May-2012	02:03:52	5.8	11.26	44.90	6.3	Govoni *et al*.^58^	2,366	12,944	HSIT	0.056	0.98	5.5	1
14	29-May-2012	07:00:03	5.6	11.07	44.84	8.1	Govoni *et al*.^58^	1,794	9,165	HSIT	0.067	0.95	5.4	1
15	29-May-2012	10:55:57	5.3	10.98	44.87	8.7	Govoni *et al*.^58^	1,149	3,715	HSIT	0.057	0.97	4.9	1
16	03-Jun-2012	19:20:43	4.7	10.95	44.89	8.7	Govoni *et al*.^58^	1,512	5,868	HSIT	0.046	0.93	4.3	1
17	06-Jun-2012	04:08:31	4.0	12.32	44.40	31.1	ISIDe	703	3,314	HSIT	0.025	0.72	4.0	7
18	25-Jan-2013	14:48:18	4.8	10.45	44.16	19.8	ISIDe	1,129	5,686	HSIT	0.015	0.81	3.8	4
19	18-Nov-2018	12:48:46	4.0	12.49	44.05	36.8	ISIDe	1,646	5,429	HSIT	0.003	0.96	3.4	7
20	14-Jan-2019	23:03:56	4.6*	12.32	44.37	20.6	ISIDe	1,748	7,425	HSIT	0.026	0.69	3.8	7

**Figure 3:**
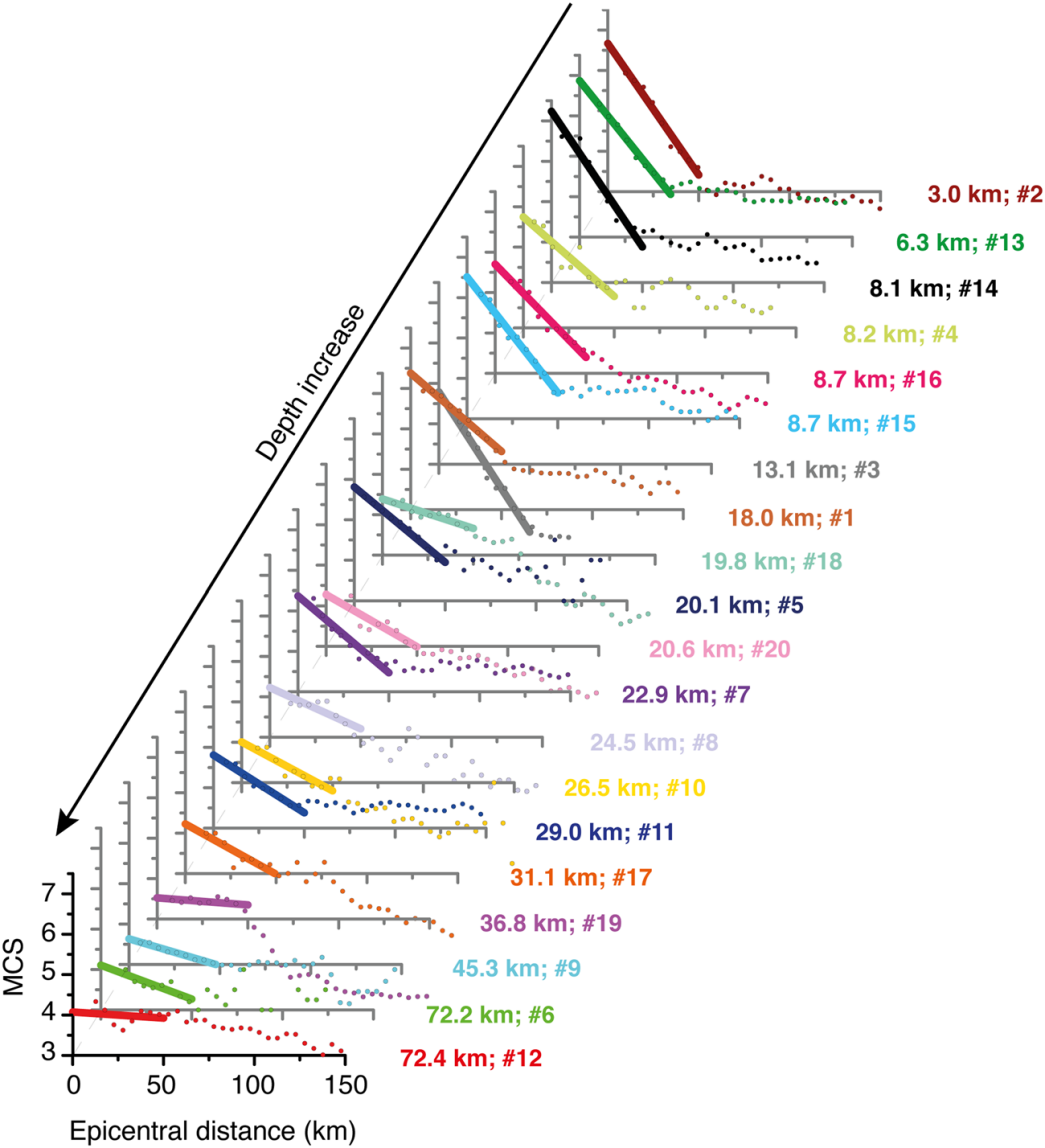
Attenuation curves obtained for all the earthquakes of the *learning set* (Fig. 1 and Table 2). Individual intensity datapoints were obtained averaging the intensity values over a circular moving window of 10 km, overlapping the adjacent window by 5 km. We used a linear fit for the first 50 km of the curves and calculated the resulting slope.

The magnitudes are all M_w_ except for events #9 and #20 (highlighted with an asterisk), for which we show an M_L_. MDP is the total number of available Macroseismic Data Points. The macroseismic data are from the ASMI database^59^ (https://emidius.mi.ingv.it/ASMI/) for the earthquakes from #1 to #5, and from the HSIT database for #6 to #20 (see Suppl. Table 1 for a direct link to the web page of each earthquake). The “total responses” is the total number of macroseismic questionnaires available for each earthquake and is shown only for the HSIT dataset. For each earthquake the slope of the attenuation curve was calculated using Eq. 1 and fitting 8 or 9 points: the results are shown in Fig. 3. The Source type column lists hypothesised types of causative faults inferred from location, depth and focal mechanism and compared with the reference tectonic model (see Fig. 1).

For each event we generated an attenuation curve based on HSIT data: given the wealth of closely-spaced datapoints, the intensity values were averaged over circular moving windows of 10 km – i.e. over progressively larger rings centred on the epicentre – each one overlapping the adjacent by 5 km: an approach similar to that used by Gasperini^2^. By doing this we averaged out any possible anisotropies, so that the observed trend can be assumed independent of local anomalies. Other investigators^4^ used a different binning method based on intensity values, referred to as “intensity binning method”. We decided not to use this approach, however, because in the first 50 km the intensity decay is generally low, especially for deeper events; this would result in a very limited number of averaged intensity points, whose linear fit would return highly uncertain results. Nevertheless, the two approaches show similar results within the first 100 km from the epicentre^4^.

Following Gasperini^2^, who showed that the first part of the intensity attenuation function has a linear trend up to 40–50 km, we calculated the parameters of the line that fits best the first 50 km of the attenuation curves of the three 2012 earthquakes discussed above. We noticed that their slope decreases as the hypocentral depth increases (see Table 2). This encouraging result led us to extend the analysis to 17 additional earthquakes that occurred in our study area, selected following the criteria described in the Method section. Together with the three 2012 earthquakes, all these events form a *learning set* to be used for subsequent elaborations. Figure 3 shows that they support the initial observation that the slope of the attenuation curve in the distance range 0–50 km correlates well with hypocentral depth. In contrast, the slope does not seem to be affected by magnitude, as clearly shown by events #10, 11 and 20, having a rather different M_w_ (4.0–4.9) but a similar depth (21.0–29.0 km), and hence a similar slope (0.024–0.029), while events #16 and 20 exhibit a similar M_w_ (4.6–4.7) but a rather different depth (8.7–20.6), resulting in a very different slope (0.026–0.046); see Table 2 and Fig. 3.

**Figure 4:**
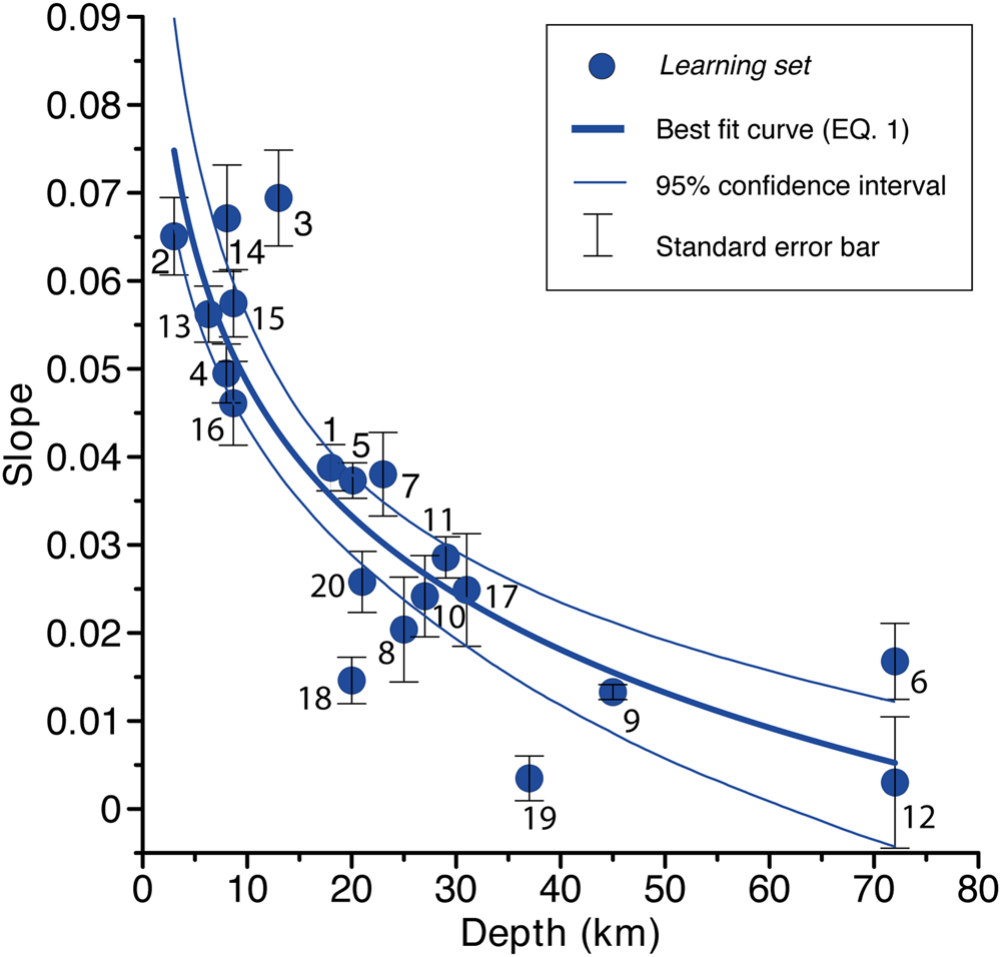
Depth versus attenuation slope for the 20 earthquakes used as a *learning set*, fitted with the logarithmic function of Eq. 1 and shown along with the 95% confidence interval. Each input value is labelled with the earthquake ID (same as Fig. 1 and Table 2) and is shown along with its standard error relative to the slope.

We then computed the absolute value of the slope (S) for the attenuation curve of each earthquake (Table 2) and plotted it versus the hypocentral depth (D). We found that the data points (Fig. 4) are fitted by the logarithmic function with a Pearson Coefficient of 0.87, corresponding to a significance level of correlation better than 10^−3^:

1 $${\rm{S}}=(-0.022\pm 0.003)\,{\rm{l}}{\rm{n}}\,{\rm{D}}+0.099\pm 0.009$$

We are now ready to use Eq. 1 as a modern *Rosetta Stone* that should reveal a plausible range for the hypocentral depth of pre-instrumental earthquakes, once the slope of their attenuation curve is calculated. The data show that this slope - and hence the entire approach - is independent of the magnitude of the earthquake being considered, but depends largely on its focal depth (Fig. 3 and Table 2).

We then selected all the earthquakes that occurred in our study areas since the year 1800 and are backed by at least 100 MDP (Fig. 1). Our selection resulted in 17 events with magnitude ranging between 4.9 and 6.5 (Table 3; events from #24 to #40). Their epicentres fall in both the extensional and compressional domains of the Northern Apennines. We also included three older earthquakes that are backed by less than 100 MDP but are among the largest ever reported in our study area (17 November 1570, M_w_ 5.4; 19 March 1624, M_w_ 5.4; and 11 April 1688, M_w_ 5.8; respectively #21, #22 and #23 in Table 3). For each of these 20 events, which we refer to collectively as the *analysed set*, we obtained the expected depth and the associated depth range (Table 3), based on the confidence intervals shown in Fig. 4.

**Table 3:** List of the historical and early-instrumental earthquakes analysed in this study (referred to as the *analysed set*) and shown in Fig. 1.

ID	Date	Time UTC	Original M_w_	Lon	Lat	MDP	Macroseismic data source	R^2^	Slope	Expected depth (km)	Depth range (km)	Recalculated M_L_	Recalculated M_w_	Source type	Original M_0_ (Nm)	Recalculated M_0_ (Nm)	% difference
21	17-Nov-1570	19:10:00	5.4	11.632	44.824	58	CFTI5Med	0.86	0.044	12	9|16	/	/	1/3	/	/	/
22	19-Mar-1624	/	5.4	11.848	44.642	18	DBMI15	0.95	0.089	2	0|3	/	/	1	/	/	/
23	11-Apr-1688	12:20:00	5.8	11.942	44.390	39	CFTI5Med	0.80	0.033	20	17|26	/	/	2/3	/	/	/
24	9-Oct-1828	2:20:00	5.7	9.047	44.821	110	CFTI5Med	0.86	0.027	26	22|34	6.3	6.1	4/7	4.78 × 10^17^	15.48 × 10^17^	224
25	14-Feb-1834	13:15:00	6.0	9.859	44.432	112	CFTI5Med	0.83	0.065	5	3|8	/	/	5	/	/	/
26	17-Mar-1875	23:51:00	5.5	12.659	44.209	144	CFTI5Med	0.38	0.024	31	25|39	6.1	5.8	3/7	2.57 × 10^17^	7.24 × 10^17^	182
27	4-Mar-1898	21:05:00	5.4	10.260	44.655	313	CFTI5Med	0.81	0.054	8	6|11	4.7	4.3	1	1.43 × 10^17^	0.04 × 10^17^	−97
28	26-Jun-1899	23:17:22	5.0	11.117	43.958	138	CFTI5Med	0.84	0.060	6	4|9	/	/	5	/	/	/
29	13-Jan-1909	0:45:00	5.4	11.688	44.579	867	DBMI15	0.81	0.017	41	34|56	6.4	6.2	3	1.38 × 10^17^	23.44 × 10^17^	1,598
30	19-Feb-1911	7:18:30	5.3	12.074	44.117	181	CFTI5Med	0.82	0.052	9	7|12	4.7	4.3	1	0.98 × 10^17^	0.03 × 10^17^	−96
31	27-Oct-1914	9:22:00	5.6	10.598	43.912	660	DBMI15	0.91	0.029	24	20|30	6.6	6.4	4	3.51 × 10^17^	45.19 × 10^17^	1,188
32	17-May-1916	12:20:00	5.8	12.748	44.119	132	CFTI5Med	0.92	0.055	7	6|11	6.1	5.8	1	6.76 × 10^17^	6.31 × 10^17^	−7
33	16-Aug-1916	7:06:14	5.8	12.737	44.019	257	CFTI5Med	0.94	0.056	7	5|11	6.1	5.7	1	6.76 × 10^17^	5.13 × 10^17^	−24
34	10-Nov-1918	15:12:28	6.0	11.933	43.918	188	CFTI5Med	0.97	0.071	4	3|6	/	/	5	/	/	/
35	29-Jun-1919	15:06:13	6.4	11.482	43.957	565	CFTI5Med	0.75	0.036	18	14|23	5.9	5.6	5	46.77 × 10^17^	3.05 × 10^17^	−93
36	7-Sep-1920	5:55:40	6.5	10.278	44.185	750	CFTI5Med	0.99	0.057	7	5|10	6.7	6.5	5	78.52 × 10^17^	61.66 × 10^17^	−21
37	20-Apr-1929	1:10:00	5.4	11.150	44.481	109	DBMI15	0.88	0.058	6	5|10	5.5	5.2	1	1.38 × 10^17^	0.74 × 10^17^	−46
38	15-May-1951	22:54:00	5.2	9.620	45.226	179	DBMI15	0.98	0.016	44	34|59	6.2	5.9	3	0.72 × 10^17^	9.23 × 10^17^	1,188
39	15-Jul-1971	1:33:23	5.5	10.345	44.814	228	CFTI5Med	0.92	0.050	9	8|13	6.0	5.9	1	2.32 × 10^17^	8.04 × 10^17^	247
40	25-Oct-1972	21:56:11	4.9	9.865	44.503	198	DBMI15	0.52	0.016	45	36|59	5.9	5.6	6/7	0.25 × 10^17^	3.39 × 10^17^	1,234

The *Expected depth* and the *Depth range* were calculated using Eq. 1. The M_L_ and M_w_ were recalculated using Eq. 2 and the conversion equation by Grünthal and Wahlstrom^42^. The last column shows the % increase or decrease of the recalculated seismic moment with respect to the original estimate. MDP is the number of the Macroseismic Data Points, i.e. the number of municipalities with an intensity assigned. The Source type column lists hypothesised types of causative faults inferred from location and expected depth and compared with the reference tectonic model (see Table 1 and Fig. 1).

Once the expected depth was estimated, we calculated the expected M_L_ for all the earthquakes of the *analysed set* by inverting for magnitude the Intensity Prediction Equation (IPE) proposed by Tosi *et al*.^39^. This equation, derived using the whole HSIT macroseismic dataset, takes the following form (Eq. 2):

2 $${\rm{I}}=(-2.15\pm 0.03)\log \,{\rm{r}}+(1.03\pm 0.01){{\rm{M}}}_{{\rm{L}}}+2.31\pm 0.04$$

where I is the Mercalli-Cancani-Sieberg (MCS) macroseismic intensity and r is the hypocentral distance in km. We used the average intensity data following standard methodologies^40,41^ in the distance range 0–200 km. We did not recalculate M_L_ for the events whose macroseismic field does not extend to 200 km. All the inferred M_L_ were then converted into M_w_ using the equation proposed by Grünthal and Wahlström^42^ (Table 3), to allow them to be compared with the magnitudes reported in CPTI15. We estimated the associated error by calculating the M_w_ for the *learning set* with the same procedure used for the *analysed set*. We then compared the results with the instrumentally-determined magnitudes, obtaining a root mean square error of 0.44 for the whole dataset and of 0.25 for the earthquakes of M_w_ ≥ 4.8, that is the starting magnitude of the range considered in the *analysed set* (Table 3).

## Discussion

### Characteristics of the attenuation curves

To obtain the attenuation curves we first assumed isotropic intensity attenuation, then binned intensity values inside circular windows. The finding that the attenuation curve can be approximated by a straight line in the first 50 km from the epicentre (Figs 2 and 3) agrees with the results obtained by Gasperini^2^. After imposing a hypocentral depth of 10 km for all the analysed historical earthquakes, this investigator found empirically that the attenuation of seismic intensity in Italy is well described by a bilinear model; the change in slope occurs rather abruptly between 40 and 50 km from the assumed epicentre, and the average slope of the first part of the attenuation curve is 0.05 to 0.06^2^. Previous workers observed an abrupt change in the slope of the attenuation curve at about the same epicentral distance using a numerical simulation of the attenuation of PGA^43^.

A previous investigation of seismic intensity attenuation in Italy^44^ found a correlation between the heat flow and the slope of the attenuation curve over the first 45 km from the source. In this investigation the hypocentral depth for all earthquakes was fixed at 10 km; the results show larger attenuation in areas of high heat flow. This result can be also interpreted in terms of variations of the focal depth, since the seismogenic layer is thinner in high heat flow areas^45^, and hence earthquakes are necessarily shallower.

### The *Rosetta Stone*: pros and cons

**Figure 5:**
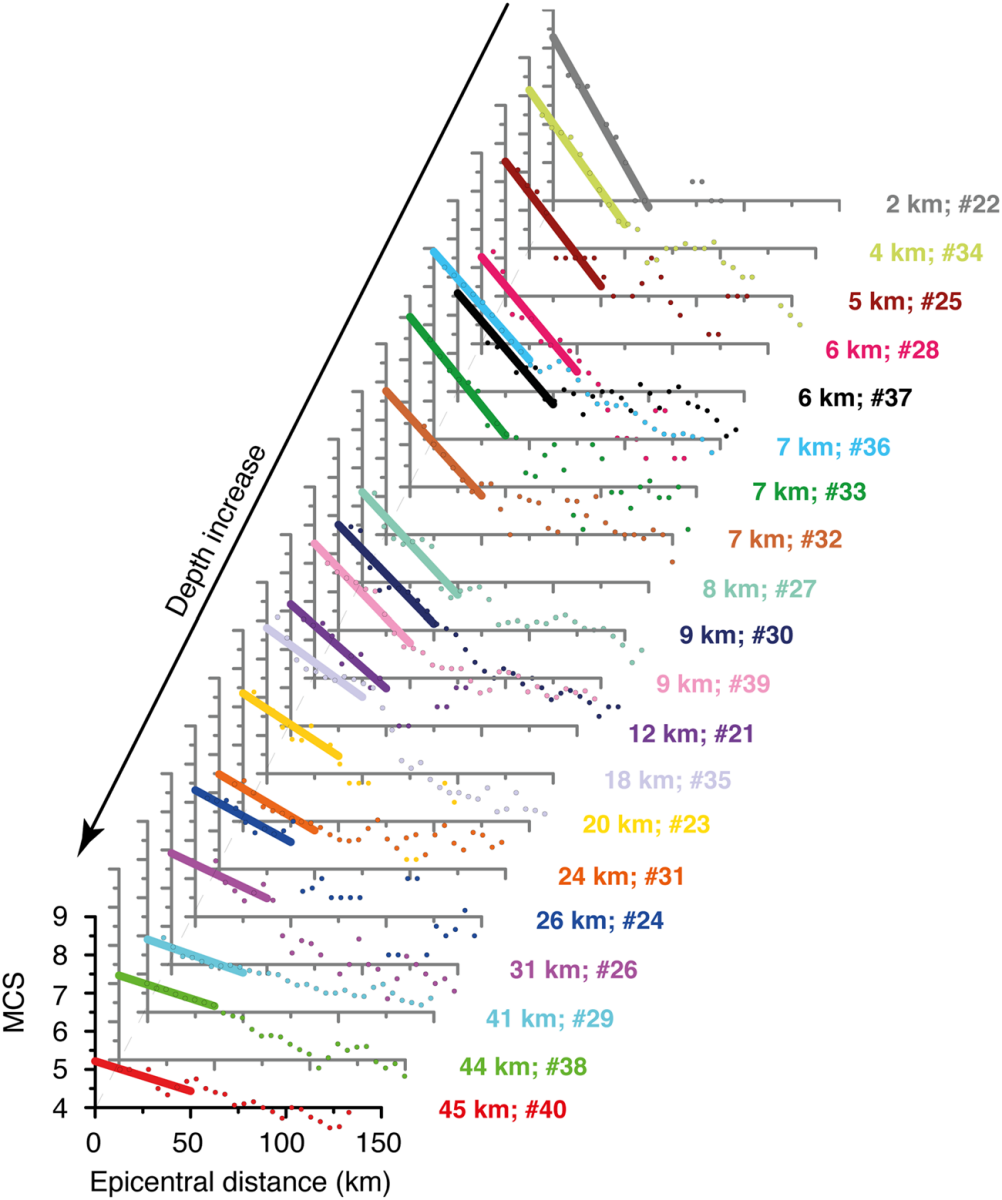
Attenuation curves obtained for all the earthquakes of the *analysed set* (Fig. 1 and Table 3). The 50-km slopes were calculated using the same procedure described for the *learning set earthquakes* (see text and Fig. 3).

Our method is independent of magnitude over the full range analysed in this study (M 4.0–6.5). In fact, we observed that the slope of the attenuation curve within the first 50 km from the epicentre is strongly influenced by the hypocentral depth, while an increase in magnitude only causes its upward shift: this circumstance results from a generally larger epicentral intensity (I_0_), and consequently larger intensity values at least within the first 50 km from the epicentre (Figs 3 and 5).

The independence of magnitude is a crucial feature that distinguishes our approach from other methods. Recently, Traversa *et al*. developed a methodology based on an exploration-tree approach to estimate the M_w_ and depth of French historical earthquakes^41^. In this procedure, however, the two unknowns, i.e. depth and magnitude, were not treated independently. On the contrary, our approach does not require calculating the magnitude, but its only requirement is an even distribution of MDP within the first 50 km around the epicentre. For example, we were able to infer the depth of the 1570, 1624 and 1688 earthquakes, the three oldest events of our dataset (Table 3), despite the limited number of available MDPs; but we are well aware that the poor data coverage inevitably translates into a larger uncertainty in slope estimation (see the Method section for a description of the estimation procedure).

The inferred depth range of the *analysed set* (Table 3) is larger for deeper events. This is inherently due to the logarithmic behavior of the data trend as confirmed by the log-character of the best fit curve. For example, the 1920 earthquake (#36) exhibits a slope of 0.057, implying that it is a shallow event with a depth range of 5 to 10 km and an expected depth of 7 km, while the 1909 earthquake (#29), whose slope is 0.017, is a deeper event falling in the range 34 to 56 km and having a preferred depth of 41 km. Hence, all shallow earthquakes, i.e. those whose slope and depth are >0.045 and <15.0 km, respectively (Fig. 4), are easier to discriminate from all other intermediate and deep events, whereas separating the deepest events is not straightforward. In spite of this limitation, even a raw depth estimation is usually sufficient to assign these deeper earthquakes to their relevant source type (Fig. 1), given the large difference between the minimum and maximum depth of seismogenic faults in our study area^1,20^ (Table 1).

### Applying the *Rosetta Stone* to selected unknown-depth earthquakes: seismotectonic implications

Here we discuss the relationships between the hypocentral depth we calculated using Eq. 1 for earthquakes of the *analysed set* (Table 3) and their relevant seismogenic source types (#1, #3, #4 and #7 for the Po Plain; #2 to #7 for the hinge of the Apennines: see Fig. 1 and Table 1).

For some of the earthquakes located in the Po Plain (#21, #23, #26, #29 and #38 in Fig. 1 and Table 3) we obtained a depth that falls well below the southward-dipping basal detachment of the outer Northern Apennines thrust fronts, locally attested between 5 and 15 km depth^14^ (Fig. 1). We also recalculated the magnitude of these earthquakes in view of their estimated hypocentral depth, with the exception of the 1570 and 1688 events because their MDPs do not reach a distance of 200 km from the epicentre.

Some of the results we obtained are quite striking: for example, the recalculated M_w_ for the deep 1909 earthquake (#29 in Table 3) is 6.2: a figure that, even considering the associated mean root square error of 0.25 obtained from the *learning set*, is significantly larger than the 5.4 proposed by the CPTI15 catalogue^18^. Altogether these results highlight the significant seismogenic potential of the inherited faults that occur in the Adriatic crust (type #3 in Fig. 1). In the literature some of the earthquakes they generated have been associated with shallow thrust faults^1^ (e.g. the 1688 and 1875 events), but based on the new depth estimation they should now be reinterpreted as belonging to one of the deeper domains. More specifically, the 1909 earthquake, that had already been hypothesised to have a deep source^46^, falls close to the epicentre of the M_w_ 5.5, 1796 earthquake, whose macroseismic field resembles that of a deep earthquake^20^. Unfortunately, the number of MDPs available for this event is too limited for it to be analysed in this study; nevertheless, we suggest that also this earthquake has a deep source, and hence that its magnitude is likely to be even larger than that recalculated for the 1909 earthquake.

Moving westward, for the 1951 earthquake (#38 in Fig. 1 and Table 3) we found an estimated depth range confirming this as a deep event, in agreement with a recent reappraisal based on instrumental data^19^. The occurrence of this earthquake has long been debated as its hypocentre falls below a major gas- and oil-field that started being exploited after World War II. For this reason, it was originally suggested it could have been triggered by gas extraction^47^, implying a shallow source. Conversely, our elaborations put its hypocentre in a depth range 36–59 km (Table 3), suggesting that the earthquake was caused by a still unidentified inherited fault in the lower Adria crust (type #3 in Fig. 1) and dismissing the hypothesis that it was triggered by human activities. Its recalculated M_w_ is 5.9 ± 0.25, significantly larger than the M_w_ 5.2–5.3 respectively assigned by the CPTI15 catalogue^18^ and by the instrumental determination^19^.

For other earthquakes that occurred in the study area (events #22, #27, #30, #32, #33, #37 and #39: Fig. 1 and Table 3) we found a shallow hypocentral depth that relates them to the activity of the outer Northern Apennines thrust fronts (type #1), in agreement with previous regional and local studies^1,20,48,49^; this stresses even further their seismogenic potential, recently highlighted by the May 2012 earthquake sequence.

Although the instrumental earthquakes used to build the curve of Fig. 4 were generated mostly by shallow and mid-crustal compressional sources of the Northern Apennines thrust belt (Table 2), since our analysis did not take into account the faulting mechanism, we applied our method also to earthquakes that occurred along the crest of the Apennines, such as the 29 June 1919, M_w_ 6.4, Mugello and the 7 September 1920, M_w_ 6.5, Garfagnana events (#35 and #36 in Table 3). These are the largest earthquakes of the whole dataset, and due to their location, they were likely generated by shallow crustal extensional faults^1^ (type #5 in Fig. 1) belonging to the northern Etrurian Fault^21^, a large system of gently northeast-dipping normal faults that typically extend up to 20 km depth.

For the 1920 earthquake our elaborations return a possible depth range of 5 to 10 km, fully compatible with the existing seismotectonic model^1^. Conversely, for the 1919 event we obtained a depth range 14–23 km, slightly deeper than expected. The macroseismic field of this earthquake, however, is known to have been contaminated by the effects of the 10 November 1918, M_w_ 6.0, Santa Sofia earthquake^34^ (#34 in Table 3), which returned a depth range of 3 to 6 km and is located about 30 km east of the 1919 epicentre. On the one hand, the reported intensity field for the 1919 earthquake appears much more spread out than it is in reality, due to the difficulty of separating the effects of the previous earthquake; this results in an apparent lower attenuation of macroseismic intensity, and hence in anomalously deeper depth. On the other hand, the effects of the 1918 earthquake probably affected also our calculation of the magnitude of the 1919 earthquake, since the M_w_ 5.6 we obtained is definitely smaller than the M_w_ 6.4 supplied by the CPTI15 catalogue. Similar contaminations are common in Italy as much as in many other seismogenic areas and call for special care when dealing with earthquakes whose territorial impact overlaps with that of nearby, similarly large shocks.

### Potential seismic hazard implications

Our findings have important implications for seismic hazard assessment, especially when conducted at the local scale and in areas of low or sparse seismicity. Under these circumstances any SHA analysis, either deterministic or probabilistic, usually relies on a handful – sometimes just one or two – of early- or pre-instrumental earthquakes, representing the only available source of seismological information. This makes it crucial to characterise them as accurately as possible based on their intensity reports.

The focal depth is indeed a most crucial parameter for all these analyses, because:

- earthquakes occurring at different depth intervals can be assigned to different seismotectonic categories (see Table 1 and inset in Fig. 1), which allows also their kinematics and their expected rates of occurrence to be determined with better confidence. This information and the ensuing hypotheses may eventually lead to devise a 3D model of seismogenic zonation^46^ – i.e. a model that is different at different depths – thus overcoming a known limitation of standard zonation models^50^;
- the expected increase of the inferred magnitude with depth may also have substantial seismic hazard implications. We find that in some instances the corresponding M_0_ increases by over one order of magnitude (see the last column in Table 3), implying that current catalogues may underestimate the earthquake budget associated with a specific type of earthquake sources by 90% or more;
- the combined increase in earthquake magnitude and depth also has substantial earthquake engineering implications, for at least two reasons: (a) a larger shock implies larger expected ground shaking, or at least a similar shaking level over a much broader region; and (b) the seismic radiation associated with larger and deeper earthquakes is generally characterised by rather different spectral amplitudes with respect to shallower and smaller events. In particular, they may feature a larger low-frequency content, which may cause resonance effects in tall buildings^51^.

## Conclusions

We elaborated a methodology to estimate the earthquake hypocentral depth based on macroseismic intensity data. The methodology is especially suited for obtaining a realistic depth range for earthquakes that occurred in the pre-instrumental and early-instrumental era, up to the 1970s, and for which there exists a detailed reconstruction of the macroseismic field. In other words, while reasonably accurate estimates of the focal depth exist for all significant Italian earthquakes of the past 50 years, our method can be used to extend this interval to the past three centuries and beyond. Hence, the method allows each earthquake to be assigned to one of the various seismogenic source types that exist at different depth intervals in any active region, providing crucial insight to improve the understanding of seismogenic processes and of their rates.

By analysing the slope of the first 50 km of the attenuations curve of well-documented recent earthquakes we found a statistically significant correlation with their hypocentral depth. The correlation obtained from this *learning set* is the key to infer the depth range of historical and early-instrumental earthquakes. Our methodology was tested on a set of such earthquakes - which we referred to as the *analysed set* - that occurred in northern Italy, a region characterised by multiple seismogenic sources occurring at different depth. The test returned encouraging results and our methodology is ready to be extended to any other seismogenic area of the world, ideally following a re-calibration based on local data.

The method is easy to use, independent of magnitude, and suitable also for incomplete macroseismic intensity fields, as in the case of the oldest earthquakes of our dataset, dating back to the 16^th^ and early 17^th^ century. We wish to stress that the method works independently of magnitude, implying that the obtained depth estimates can be used to recalculate the macroseismic magnitude itself.

We show that for earthquakes deeper than 30 km the revised magnitudes are consistently larger than those based on intensity alone (e.g. using the Boxer code). We conclude that in addition to highlighting the existence of deeper and less known seismogenic sources, our methodology may have a significant impact on the calculation of annual earthquake rates, and hence on seismic hazard assessment, particularly in areas of infrequent seismicity and low strain rates.

## Method

### Selection criteria for the *learning set*

We selected the events from the web-based macroseismic HSIT catalogue (Fig. 1; #6 to #20 in Table 2), that relies on the INGV catalogue^52^ for instrumental earthquake locations, using the following criteria:

- M ≥ 4.0 for earthquakes deeper than 25 km, in order to collect a statistically significant number of events;
- M > 4.5 for events having hypocentral depth ≤25 km.
  From this large dataset we discarded:
- all the events whose hypocentral depth was fixed (i.e. not obtained from the seismograms);
- all the events for which less than 1,000 questionnaires are available;
- all the aftershocks that occurred too close in time to the mainshock, in order to prevent any possible mistakes by the compilers^53^.

These rather strict criteria were fulfilled by 14 earthquakes, including the three 2012 earthquakes discussed earlier and shown in Fig. 2, ranging in depth from 3.0 to 72.4 km and ranging in M_w_ between 4.0 and 5.8 (Table 2). To further extend our dataset we also included six significant earthquakes that do not fit the above requirements:

- the 26 March 2008, M_w_ 4.0 earthquake (#6 in Table 2): a relatively small event that is backed by only 85 questionnaires but occurred at 72 km depth and is well constrained by seismological data. This event is crucial for extending the explored focal depth range, being one of the only two events that occurred at a depth of over 50 km;
- five additional earthquakes falling in our study area but not included in the HSIT catalogue because they occurred before 2007 (Fig. 1), provided that their location is reliable and, more specifically, that their hypocentral depth is calculated (non-fixed).

These latter five events (#1 to #5 in Table 2) were taken from the DBMI15^37^ and CFTI5Med^34^ databases; they all have M > 4.5 and a number of MDP larger than 100. We made this choice following the results of a recent study^54^ that has shown that traditional macroseismic datasets are fully consistent with HSIT observations.

Unfortunately our learning set, that is the outcome of the application of these selection criteria, exhibits an uneven distribution of earthquakes with depth, the events shallower than 35 km being 80% of the total.

### Intensity estimation methods

The macroseismic data of recent earthquakes that occurred since 2007 were obtained through an online macroseismic questionnaire “Hai Sentito Il Terremoto” (*Did You Feel the Earthquake*), developed, published and maintained by INGV (http://www.haisentitoilterremoto.it/). The online questionnaire is compiled by volunteers and by registered users (currently about 27,000, spread over the whole Italian territory). Each questionnaire is analysed through automatic procedures, and a macroseismic intensity is ultimately assessed for each municipality. The procedure for the interpretation of the questionnaires was described in detail in previous papers^36,39^. The HSIT macroseismic intensities have been found to be in good agreement with the intensity values obtained from conventional macroseismsic surveys^36,39,54^.

The macroseismic data for earthquakes that occurred before 2007 were derived from field macroseismic surveys or from the interpretation of questionnaires compiled by Italian local administrations. Macroseismic data of recent and historical earthquakes reported in the CPTI15 database^18^ derive from as many as 185 original studies, databases, reports, and bulletins. In particular, the data for most of the largest earthquakes were supplied by the CFTI5Med catalogue^34^, a new-generation analytical catalogue that reports a great deal of information on the effects of each earthquake at each of the affected localities. Tables 2 and 3 report the source of macroseismic data for each earthquake analysed in this work.

### Reference databases for macroseismic data

- HSIT macroseismic data (Table 2) are available at http://www.haisentitoilterremoto.it/ (last accessed October 2019).
- Pre-2007 and historical macroseismic data (Tables 2 and 3) are available from the following databases:

- ASMI catalogue: https://emidius.mi.ingv.it/ASMI^4^ (last accessed October 2019).
- CFTI5Med: http://storing.ingv.it/cfti/cfti5/^34^ (last accessed October 2019).

See Supplementary Tables 1 and 2 to find out the URL of the web page containing the macroseismic data available for each earthquake.

### Uncertainty estimation of the slope of the attenuation curves

The reliability of the slope calculated for the initial 50 km of the attenuation curves, both for recent and for historical earthquakes, is controlled by several factors, including the anisotropy of attenuation, the number of available MDPs, and the intrinsic uncertainties in MDPs.

The attenuation curves are obtained fitting averaged intensities in a circular moving window of 10 km, overlapping the adjacent window by 5 km. The slope of the attenuation curves in the first 50 km is calculated from a first-degree fit of a set of 8–9 averaged intensities, depending on data availability. The resulting coefficient of determination R^2^ values are often good at a significance level better than 10^−3^, except for earthquakes #6, 8, 17, 20, 23, 35, that exhibit a significance of 10^−2^. Earthquakes #12, 26, 40 exhibit a scattered attenuation pattern, causing low R^2^ (Tables 2 and 3). Each slope value is supplied along with its standard error bar (Fig. 4).

### Earthquake parameters

The parameters of the earthquakes of the *learning set* that occurred since 1985 were obtained from the INGV earthquake catalogue^52^ (available from http://cnt.rm.ingv.it/iside) and are listed in Table 2. The parameters of the only event older than 1985 (#1) were derived from the CSTI1.1 database^55^. For the depth values we used data from the same databases, except when there exists a specific study about a given earthquake; in that case, we preferred the re-located depth given in the study (source specified in Table 2). The parameters of the *analysed set* (Table 3) were taken from the CPTI15 and CFTI5Med catalogues^18,34^.

## Supplementary information

Supplementary file

## Data Availability

All macroseismic data used during this study can be downloaded from public web sites (see Supplementary Tables 1 and 2 for details).
