# Supplementary material for: Inferring the depth of pre-instrumental earthquakes from macroseismic intensity data: a case-history from Northern Italy
Source: Sci Rep. 2019 Oct 30;9:15583. doi: 10.1038/s41598-019-51966-4 (PMC6821853; doi:10.1038/s41598-019-51966-4)
Supplement: Supplementary file 1 — Supplementary file [file 41598_2019_51966_MOESM1_ESM.docx]

Supplementary information for

**Inferring the depth of pre-instrumental earthquakes**

**from macroseismic intensity data:**

**a case-history from Northern Italy**

Paola Sbarra*, Pierfrancesco Burrato, Patrizia Tosi, Paola Vannoli, Valerio De Rubeis and Gianluca Valensise

*Istituto Nazionale di Geofisica e Vulcanologia*

*paola.sbarra@ingv.it

**Supplementary Table 1**: table showing the link to the web page of the macroseismic data related to the individual earthquakes composing the *learning set*.

**Supplementary Table 2**: table showing the link to the web page of the macroseismic data related to the individual earthquakes composing the *analysed set*.
